# Epigenetic Alterations in Liver of C57BL/6J Mice after Short-Term Inhalational Exposure to 1,3-Butadiene

**DOI:** 10.1289/ehp.1002910

**Published:** 2010-12-13

**Authors:** Igor Koturbash, Anne Scherhag, Jessica Sorrentino, Kenneth Sexton, Wanda Bodnar, Volodymyr Tryndyak, John R. Latendresse, James A. Swenberg, Frederick A. Beland, Igor P. Pogribny, Ivan Rusyn

**Affiliations:** 1 Division of Biochemical Toxicology, National Center for Toxicological Research, U.S. Food and Drug Administration, Jefferson, Arkansas, USA; 2 Technical University of Kaiserslautern, Kaiserslautern, Rheinland-Pfalz, Germany; 3 Curriculum in Toxicology, University of North Carolina–Chapel Hill, Chapel Hill, North Carolina, USA; 4 Department of Environmental Sciences and Engineering, University of North Carolina–Chapel Hill, Chapel Hill, North Carolina, USA; 5 Toxicologic Pathology Associates, National Center for Toxicological Research, Jefferson, Arkansas, USA

**Keywords:** 1,3-butadiene, DNA damage, epigenetics, liver, mouse

## Abstract

**Background:**

1,3-Butadiene (BD) is a high-volume industrial chemical and a known human carcinogen. The main mode of BD carcinogenicity is thought to involve formation of genotoxic epoxides.

**Objectives:**

In this study we tested the hypothesis that BD may be epigenotoxic (i.e., cause changes in DNA and histone methylation) and explored the possible molecular mechanisms for the epigenetic changes.

**Methods and Results:**

We administered BD (6.25 and 625 ppm) to C57BL/6J male mice by inhalation for 2 weeks (6 hr/day, 5 days a week) and then examined liver tissue from these mice for signs of toxicity using histopathology and gene expression analyses. We observed no changes in mice exposed to 6.25 ppm BD, but glycogen depletion and dysregulation of hepatotoxicity biomarker genes were observed in mice exposed to 625 ppm BD. We detected *N*-7-(2,3,4-trihydroxybut-1-yl)guanine (THB-Gua) adducts in liver DNA of exposed mice in a dose-responsive manner, and also observed extensive alterations in the cellular epigenome in the liver, including demethylation of global DNA and repetitive elements and a decrease in histone H3 and H4 lysine methylation. In addition, we observed down-regulation of DNA methyltransferase 1 (Dnmt1) and suppressor of variegation 3–9 homolog 1, a histone lysine methyltransferase (Suv39h1), and up-regulation of the histone demethylase Jumonji domain 2 (Jmjd2a), proteins responsible for the accurate maintenance of the epigenetic marks. Although the epigenetic effects were most pronounced in the 625-ppm exposure group, some effects were evident in mice exposed to 6.25 ppm BD.

**Conclusions:**

This study demonstrates that exposure to BD leads to epigenetic alterations in the liver, which may be important contributors to the mode of BD carcinogenicity.

The gaseous olefin 1,3-butadiene (BD) is a major high-volume industrial chemical monomer widely used in the production of synthetic rubber, resins, and plastics (Himmelstein et al. 1997; White 2007). The International Agency for Research on Cancer (IARC 2008) has recognized BD as “known to be carcinogenic to humans.” In rodents, BD causes tumor formation at several sites, including the hematopoietic system, lungs, heart, and liver (Himmelstein et al. 1997; Melnick and Sills 2001). BD is metabolized in the liver through oxidation by the family of cytochrome P450 monooxygenases, a pathway that forms several epoxides, specifically 1,2-epoxy-3-butene, 1,2,3,4-diepoxybutane, and 3,4-epoxy-1,2-butanediol (Filser et al. 2007; Himmelstein et al. 1997). The genotoxicity of BD-derived epoxides is considered to be a critical event in the initiation of tumorigenesis (Cochrane and Skopek 1994; Kemper et al. 2001).

Most of the research on the carcinogenicity assessment of BD has focused on the formation of DNA adducts that lead to mutations and chromosomal aberrations (Goggin et al. 2009; Swenberg et al. 2000); however, even within classical genotoxic carcinogenesis models, the formation of DNA adducts is not the only mode of carcinogenesis. Recent data point to epigenetic alterations as important adverse biological effects caused by exposure to numerous environmental chemical and physical agents (Reamon-Buettner et al. 2008). Interestingly, data from human and animal studies have demonstrated that many early indicators of exposure to environmental toxicants are epigenetic in nature (Baccarelli and Bollati 2009). It has been also suggested that epigenetic alterations, including whole-genome– and repetitive-element–associated hypomethylation and hypermethylation of the promoters of key genes (e.g., *O*^6^-methylguanine-DNA methyltransferase), may precede and/or provoke genetic alterations (Jacinto and Esteller 2007; Sawan et al. 2008). Furthermore, epigenetic changes not only may be important for understanding the molecular underpinnings of environmentally related disease but also may serve as biomarkers in toxicity and/or carcinogenicity assessment (Marlowe et al. 2009; Reamon-Buettner et al. 2008).

It is not clear whether exposure to BD causes epigenetic alterations in target tissues in addition to the induction of well-defined genotoxic changes. Thus, we tested the hypothesis that BD is also epigenotoxic (i.e., causes changes in DNA and histone methylation) and we explored the possible molecular mechanisms of these effects.

## Materials and Methods

### Animals and experimental design

Male C57BL/6J mice (Jackson Laboratory, Bar Harbor, ME, USA) were housed in sterilized cages in a temperature-controlled (24°C) room, with a 12/12-hr light/dark cycle, and given *ad libitum* access to purified water and NIH-31 pelleted diet (Purina Mills, Richmond, IN, USA). After a 2-week acclimation period, the mice (9 weeks of age) were allocated randomly into three groups (*n* = 5 per group): one control group (exposed to filtered air) and two experimental groups (exposed to 6.25 ppm or 625 ppm BD). Exposures were conducted 6 hr/day, 5 days/week (Monday through Friday) for 2 weeks. Each experimental day, mice were placed in a cylindrical metal mesh holder for the duration of exposure and then returned to their cages. The concentrations of BD in exposure chambers were monitored before and after each exposure period using gas chromatography and determined to correspond to the target concentrations (data not shown). After the last exposure, mice were euthanized by exsanguination after deep anesthesia with isoflurane. The livers were excised, and a slice of the medial lobe was fixed in 10% neutral buffered formalin for 48 hr for histopathological examination using hematoxylin and eosin–stained sections. The remaining liver was frozen immediately in liquid nitrogen and stored at −80°C for subsequent analyses. The animals were treated humanely and with regard for alleviation of suffering. The experiments were approved by the Institutional Animal Care and Use Committee at the University of North Carolina at Chapel Hill.

### Determination of N-7 guanine adduct formation

Genomic DNA was isolated from mouse liver tissues by standard digestion of the tissue with proteinase K, followed by phenol-chloroform extraction and ethanol precipitation. Levels of *N*-7-(2,3,4-trihydroxybut-1-yl)guanine (THB-Gua) were analyzed by liquid chromatography/tandem mass spectrometry as described by Koc et al. (1999) with minor modifications. Briefly, DNA (300 μg) from each sample was spiked with 500 fmol THB-Gua internal standard and adjusted with distilled water to 400 μL volume. Before injection into the ACQUITY UPLC column (Waters Corp., Milford, MA, USA) coupled to TSQ Quantum Ultra mass spectrometer (Thermo Fisher Scientific, Waltham, MA, USA), neutral thermal hydrolysis was performed at 95°C for 30 min and samples were centrifuged through a 10-kDa filter twice.

### Quantitative reverse-transcription polymerase chain reaction (PCR)

Total RNA was extracted from mouse liver tissues using TRI reagent (Ambion, Austin, TX, USA) according to the manufacturer’s instructions. cDNA was synthesized from 5 μg total RNA using the RT^2^ First Strand cDNA synthesis kit (SABiosciences, Frederick, MD, USA). We used Mouse Hepatotoxicity RT^2^ Profiler PCR Arrays (SABiosciences), according to the manufacturer’s protocol, to determine expression of 84 key genes implicated as potential biomarkers of liver toxicity. The relative level of mRNA for each gene was determined using the 2^ΔΔCt^ method (Schmittgen and Livak 2008). The results are presented as fold change for each mRNA in the liver of mice exposed to BD relative to those from control mice.

### Determination of global DNA methylation status by methylation-sensitive cytosine extension assay

We evaluated the extent of global DNA methylation using a radiolabeled deoxycytidine-5′-triphosphate ([^3^H]dCTP) extension assay as described elsewhere (Pogribny et al. 1999).

### Methylation-sensitive quantitative PCR (qPCR) analysis of repetitive elements methylation

The methylation status of major and minor satellites, long interspersed elements 1 (LINE1), and short interspersed nuclear elements (SINE) B1 and B2 repetitive elements was determined by methylation-sensitive McrBC-qPCR assay (Martens et al. 2005). Briefly, genomic DNA (1 μg) was digested overnight with the methylation-specific restriction enzyme McrBC (New England Biolabs, Ipswich, MA, USA) and then analyzed by qPCR on an ABI 7900 Real-time PCR System (Applied Biosystems, Forrest City, CA, USA). The threshold cycle (C_t_) was defined as the fractional cycle number that surpassed the fixed threshold. The C_t_ values were converted into the absolute amount of input DNA using an absolute standard curve method. An increased amount of input DNA after digestion with McrBC was indicative of hypomethylation, whereas a decreased amount of input DNA was indicative of hypermethylation.

### Methylated DNA immunoprecipitation (MeDIP)-qPCR analysis of LINE1 methylation

We used a MeDIP assay combined with qPCR to assess quantitatively the methylation status of LINE1 repetitive sequences in liver of control and BD-exposed mice. MeDIP was performed as described by Weber et al. (2005). Briefly, 5 μg genomic DNA was randomly sheared to an average length of 0.2–1.0 kb by sonication and divided into immunoprecipitated and input portions. DNA from the immunoprecipitated portions was incubated overnight at 4°C with a monoclonal antibody (5 μg) against 5-methylcytosine (Abcam, Cambridge, MA, USA), followed by overnight incubation with Pan-mouse IgG Dynal magnetic beads (Invitrogen, Carlsbad, CA, USA) at 4°C. The methylated DNA/antibody complexes were then digested with proteinase K, and enriched DNA was recovered by phenol-chloroform extraction followed by ethanol precipitation. Purified DNA from immunoprecipitated DNA and input DNA samples were analyzed by qPCR on an ABI 7900 real-time PCR system as described above. The relative changes in the extent of LINE1 methylation were determined by measuring the amount of DNA in immunoprecipitated DNA after normalization to the input DNA.

### Western blot analysis of histone modifications and protein expression

We used the Western blot analysis procedure described by Tryndyak et al. (2006) to assess the status of histone H3 lysine 9 (H3K9), histone H3 lysine 27 (H3K27), and histone H4 lysine 20 (H4K20) trimethylation, as well as protein levels of DNA methyltransferase 1 (Dnmt1), histone lysine methyltransferases Suv39h1 and Suv4-20h1, histone demethylase Jumonji domain 2 (Jmjd2a), and β-actin. Briefly, acid extracts of total histones were isolated from the liver tissues, separated by sodium dodecyl sulfate–polyacrylamide gel electrophoresis, and subjected to immunoblotting using specific antibodies against trimethylated histone H3K9 (H3K9me3), H3K27me3, and H4K20me3. Chemiluminescence detection was performed with horseradish peroxidase substrate for Western blotting (Millipore Corp., Billerica, MA, USA) and measured directly by a BioSpectrum AC Imaging System (BioSpectrum, Upland, CA, USA). The signal intensity was analyzed by ImageQuant software (version 5.1; Molecular Dynamics, Sunnyvale, CA, USA).

### Statistical analyses

Results are presented as mean ± SD. Comparisons between control and BD-exposed mice were made by Student’s *t*-test; *p*-values < 0.05 were considered significant.

## Results

### Effects of BD exposure on body weight, serum alanine aminotransferase (ALT) levels, and liver histopathology

**Figure 1:**
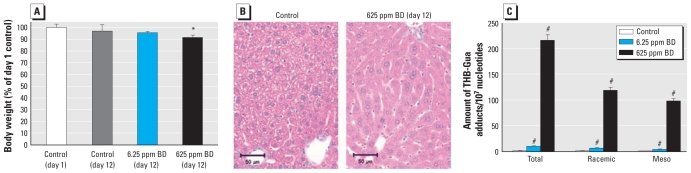
Effects of BD exposure on body weight, liver histopathology, and DNA damage. (*A*) Body weights (mean ± SD) of control mice at the beginning and end of the study and of mice exposed to 6.25 ppm BD or 625 ppm BD for 2 weeks (*n* = 5). (*B*) Representative images of hematoxylin and eosin–stained liver sections from control mice and mice exposed to 625 ppm BD (original magnification, 400×). (*C*) Amounts (mean ± SD) of THB-Gua–BD adducts (total and racemic and meso forms) in liver DNA from mice exposed to 0, 6.25, or 625 ppm BD (*n* = 5). *p < 0.05 compared with control mice at day 12. ^#^*p* < 0.05 compared with control.

We evaluated changes in body weight and serum ALT activity, and histomorphological alterations in the liver of C57BL/6J mice exposed to 0, 6.25, or 625 ppm BD. We observed a small (> 10%) yet significant body weight loss only in mice exposed to 625 ppm BD (Figure 1A). There were no significant changes in the liver sample weights between the control group and either treatment group. Serum ALT activity did not change in mice exposed to BD (data not shown). Histopathological evaluation of the liver sections revealed that the hepatocytes of control mice manifested minimal to mild hepatocellular cytoplasmic vacuolization morphologically characteristic of glycogen (Figure 1B, left). In contrast, the hepatocytes of the BD-treated mice showed diffuse glycogen depletion characterized by the lack of cytoplasmic vacuoles (Figure 1B, right).

### Effect of BD exposure on expression of hepatotoxicity-related biomarker genes

To determine whether inhalational exposure to BD resulted in the molecular changes indicative of liver toxicity, we examined expression of a panel of genes known to serve as biomarkers of toxicity. Livers from mice exposed to 6.25 ppm BD showed minimal changes in gene expression, whereas those from mice exposed to 625 ppm BD showed changes in gene expression that were more pronounced (Table 1). This was evident by a greater number of differentially expressed genes and greater magnitude of gene expression in mice exposed to 625 ppm BD compared with mice exposed to 6.25 ppm BD (Table 1). Most of the genes, including carbonic anhydrase 3 (*Car3*), leucine-rich repeat-containing G protein-coupled receptor 5 (*Lgr5*), and sterol regulatory element binding transcription factor 1 (*Srebf1*), were down-regulated (Table 1). Interestingly, decreased expression of *Car3*, *Lgr5*, and *Srebf1* genes is a common response to liver injury (Hsieh et al. 2009; Kojima et al. 2002). In contrast, the expression of heme oxygenase-1 (*Hmox1*) was up-regulated nearly 18-fold (Table 1). Hmox1 is one of several coordinately regulated proteins involved in protecting against liver injury induced by a variety of hepatotoxicants and has been suggested to be a reliable indicator of hepatotoxicity (Choi et al. 2003; Morio et al. 2006).

### Hepatic THB-Gua adduct levels

We assessed amounts of THB-Gua adducts in hepatic DNA from control mice and mice exposed to 6.25 and 625 ppm BD using mass spectrometry (Figure 1C). We observed a dose-dependent increase in THB-Gua adducts in hepatic DNA, with the levels significantly increased after both 6.25 and 625 ppm BD exposures.

### Effect of BD exposure on the levels of global DNA and repetitive elements methylation

**Figure 2:**
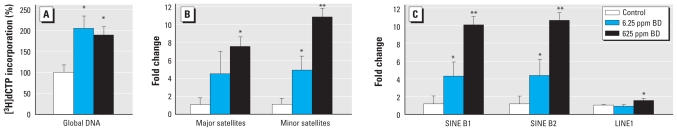
Effects of BD exposure on DNA methylation in mouse liver. (*A*) Loss of global DNA methylation in livers of BD-exposed mice measured using the cytosine extension DNA methylation assay (Pogribny et al. 1999). Methylation status of major and minor satellites (*B*) and of SINE B1 and B2 and LINE1 repetitive elements (*C*) assessed using the methylation-sensitive McrBC-qPCR assay; data are mean ± SD (*n* = 5) fold change relative to control. *p < 0.05 compared with control. **p < 0.05 compared with 6.25 ppm BD.

BD exposure resulted in substantial decreases in the levels of global DNA methylation, to similar extents in both 6.25 and 625 ppm groups (Figure 2A). The status of methylation of major and minor satellites and of LINE1 and SINE B1 and B2 repetitive elements is a sensitive indicator of the degree of global DNA methylation (Yang et al. 2004). Major and minor satellites and SINE B1 and B2 repetitive elements were demethylated in a dose-dependent manner after BD exposure (Figure 2B,C), whereas LINE1 methylation decreased only in mice exposed to 625 ppm [Figure 2C; see also Supplemental Material, Figure 1 (doi:10.1289/ehp.1002910)].

### Effect of BD exposure on histone modifications

**Figure 3:**
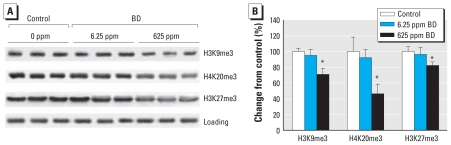
Effects of BD exposure on histone trimethylation in mouse liver as determined by Western blotting. (*A*) H3K9me3, H3K27me3, and H4K20me3 levels assessed by immunostaining using specific antibodies against trimethylated histones; equal sample loading was confirmed by immunostaining against histone H3 (“Loading” row) and histone H4 (data not shown). (*B*) Densitometry analysis of the immunostaining results shown as change in methylation relative to control after correction for the total amount of each histone in the individual samples; data are presented as mean ± SD (*n* = 5). *p < 0.05 compared with control.

Alterations in histone-controlled chromatin structure are important epigenetic changes elicited by many toxicants (Bagnyukova et al. 2008; Moggs and Orphanides 2004); therefore, we examined the effect of BD on the extent of methylation of histones H3K9, H3K27, and H4K20. We found no changes in histone trimethylation in the livers of mice exposed to 6.25 ppm BD (Figure 3). In contrast, exposure to 625 ppm BD caused a significant decrease in the levels of H3K9me3, H3K27me3, and H4K20me3: 30%, 19%, and 54% lower, respectively, than in control mice. Exposure to either 6.25 or 625 ppm BD had no effect on the extent of histone H3K9 monomethylation (H3K9me1) or dimethylation (H3K9me2) or H4K20 monomethylation (H4K20me1 [see Supplemental Material, Figure 2 (doi:10.1289/ehp.1002910)]. H4K20me2 was decreased by 39% in mice exposed to 625 ppm BD (see Supplemental Material, Figure 2).

### Effect of BD exposure on the levels of Dnmt1, Suv39h1, Suv4-20h1, and Jmjd2a proteins

**Figure 4:**
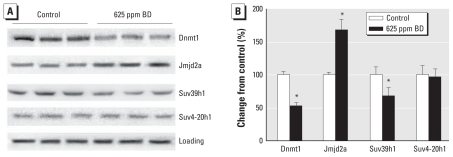
Western blot analysis of proteins responsible for DNA and histone lysine methylation in the livers of control and BD-exposed mice. (*A*) Dnmt1, Jmjd2a, Suv39h1, and Suv4-20h1 proteins assessed by immunostaining using specific antibodies; equal sample loading was confirmed by immunostaining against β-actin (“Loading” row). (*B*) Densitometry analysis of the immunostaining results shown as change relative to control; data are presented as mean ± SD (*n* = 5). *p < 0.05 compared with control.

Because BD exposure had an effect on DNA and histone methylation, we also investigated possible mechanisms of these epigenetic aberrations. Dnmt1 is the main enzyme responsible for maintaining faithful genomic methylation in somatic mammalian cells (Goll and Bestor 2005). Therefore, we assessed whether the observed loss of global DNA and LINE1 methylation in the livers of BD-exposed mice may be attributed to alterations in Dnmt1 expression. Exposure of C57BL/6J mice to 625 ppm BD resulted in a significant 47% decrease in the levels of Dnmt1 protein (Figure 4). Likewise, this concentration of BD led to a significant down-regulation of Suv39h1, the main histone methyltransferase responsible for H3K9 trimethylation (Rea et al. 2000). In contrast, levels of Suv4-20h1, a methyltransferase that catalyzes trimethylation of histone H4K20 (Benetti et al. 2007), were not altered in the livers of BD-exposed (625 ppm) mice, despite a substantial decrease in H4K20me3 (Figure 3).

To explain this discrepancy and to investigate other mechanisms that may cause the decrease in H3K9me3, we examined the levels of Jmjd2a, the histone demethylase that catalyzes demethylation of trimethylated H3K9 and H4K20 (Couture et al. 2007). Indeed, BD exposure (625 ppm) caused a significant 68% increase in the protein level of Jmjd2a in the livers (Figure 4). Levels of Dnmt1, Suv39h1, and Jmjd2a proteins did not change in mice exposed to 6.25 ppm BD (data not shown).

## Discussion

Environmental chemicals are considered to be genotoxic if they, or products of their metabolism, are shown to interact directly with DNA, causing damage and mutations and ultimately leading to tumor formation (Shuker 2002). Agents that are carcinogenic yet are not known to be DNA reactive or test negative in the genotoxicity assays are classified as nongenotoxic. Although this traditional classification has been useful in both mechanistic toxicology and regulatory decision making, mounting evidence suggests that despite differences in a chemical’s DNA reactivity, both classes of agents may lead to prominent epigenomic alterations in tissues that are targets for carcinogenesis. Recent work on epigenetic effects of various chemical agents has led to a suggestion that some carcinogens may be epigenotoxic (Pogribny et al. 2008).

The present study demonstrates that even short-term exposure of mice to BD by inhalation not only led to formation of DNA adducts but also produced prominent epigenetic changes in the liver, thus establishing BD as both a genotoxic and an epigenotoxic chemical. Specifically, we observed a pronounced loss of global DNA and LINE1 methylation; substantial decreases in histone H3K9 trimethylation, histone H4K20 dimethylation, and histone H4K20 trimethylation, and altered expression of proteins responsible for the accurate maintenance of these epigenetic marks. Even though it is well established that BD-induced cancer development involves formation of genotoxic reactive metabolites of BD and subsequent formation of DNA adducts, especially the most abundant THB-Gua adducts (Goggin et al. 2009; Koc et al. 1999), our study shows that DNA damage may not be the only mode of carcinogenic action for BD.

Several previous reports have demonstrated that exposure to chemical and pharmaceutical agents, including 2-acetylaminofluorene (Bagnyukova et al. 2008), tamoxifen (Tryndyak et al. 2006), the peroxisome proliferator WY-14,643 (Pogribny et al. 2007), ethionine (Shivapurkar et al. 1984), phenobarbital (Bachman et al. 2006), and pyrazinamide (Kovalenko et al. 2007), causes substantial epigenetic alterations in tissues that are targets for carcinogenesis. Likewise, the data presented here demonstrate that exposure to BD is associated with extensive epigenetic alterations in the liver.

One of the most pronounced changes observed in the present study was BD-induced loss of global DNA and LINE1 methylation. Several mechanisms may be responsible for global DNA demethylation, including *a*) formation of adducts with 2′-deoxyguanosine, especially in GC-rich DNA domains; *b*) DNA damage and repair; and *c*) altered expression of the main cellular DNA methyltransferase Dnmt1. Indeed, BD exposure induced profound DNA damage, especially the formation of *N*-7 guanine adducts, whose presence may profoundly compromise the methylation capacity of Dnmt1, as has been observed with other guanine adducts (Valinluck and Sowers 2007). Additionally, activation of repair-mediated DNA demethylation (Barreto et al. 2007) and decreased expression and/or functioning of Dnmt1 caused by a number of factors, including direct effects of BD and its metabolites on Dnmt1 protein, aberrant expression of microRNAs (e.g., miR-29b, miR-148, and miR-152), and expression of chromatin-modifying proteins (Garzon et al. 2009; Huang et al. 2010; Vire et al. 2006; Wang et al. 2009), may further contribute to the loss of DNA methylation.

BD-induced hypomethylation of DNA was also accompanied by a decrease in trimethylation of histones H3K9, H3K27, and H4K20. The accurate maintenance of the status of chromatin methylation depends on proper functioning and interdependent cooperation between DNA methylation and histone methylation machinery. For instance, direct interaction between Dnmt1 and Suv39h1 histone methyltransferase coordinates both DNA and histone lysine methylation (Esteve et al. 2006; Fuks et al. 2003). Because we observed down-regulation of Dnmt1 and Suv39h1 after exposure to 625 ppm BD, these proteins may not only induce demethylation of DNA but also lead to a decrease in histone lysine trimethylation. Correspondingly, expression of Jmjd2a, an enzyme that possesses lysine demethylase activity with specificity to H3K9 (Yamane et al. 2006), was up-regulated, which also may be one of the factors explaining the mechanisms for the observed effects of BD exposure on histones. Finally, the BD epoxides may also bind to methylation or acetylation sites on H3 and H4 so that they cannot be methylated or acetylated. This phenomenon has been shown for formaldehyde (Lu et al. 2008); however, the relevance of this mechanism for BD needs to be established.

Importantly, altered expression and/or functioning of Dnmt1, Suv39h1, Suv4-20h1, and Jmjd2a proteins has been attributed to destabilization of chromatin assembly and genome stability (Dion et al. 2008; Fodor et al. 2006; Peng and Karpen 2009; Peters et al. 2001). Specifically, it has been demonstrated that loss of histone H3K9, H3K27, and H4K20 trimethylation markedly impairs chromatin structure, diminishes the ability of the cells to regulate and maintain accurately the cell cycle, disrupts the balance between cell proliferation and differentiation, and severely reduces cell viability (Rea et al. 2000; Yang and Mizzen 2009). H4K20 dimethylation plays a crucial role in DNA repair by attracting the checkpoint protein Crb2 to the sites of DNA damage (Greeson et al. 2008). In addition, a decrease in H4K20me2 impairs cell survival after genotoxic insult and disrupts the ability of cells to maintain checkpoint-mediated cell-cycle arrest (Sanders et al. 2004).

## Conclusions

This study shows that short-term inhalational exposure of mice to the genotoxic carcinogen BD, in addition to inducing the formation of DNA adducts, also caused prominent epigenetic alterations in the liver. These results highlight the significance of epigenetic events in the mechanism of BD toxicity and carcinogenicity. Epigenetic alterations are not only important features of cancer cells but also play a major role in the etiology of cancer. Furthermore, alterations in the cellular epigenome, which may be termed epigenotoxicity, elicited by various genotoxic and nongenotoxic agents may result in the emergence of epigenetically reprogrammed proliferating cells with a growth-advantage phenotype and a high potential for activation of mutator pathways (Pogribny et al. 2008). We also posit that early appearance of epigenetic alterations and their association with altered expression of genes that are indicators of liver toxicity strongly suggest that changes in DNA and histone methylation may be useful predictive markers for safety assessment.

## Figures and Tables

**Table 1:** Genes significantly differentially expressed (> 2-fold) in mouse liver after BD exposure compared with control animals (*p* < 0.05).

		BD exposure (ppm)	
Gene symbol	Gene name	6.25	625
Up-regulated genes			
*Hmox1*	Heme oxygenase-1	1.2 ± 0.62	17.6 ± 4.96*
*Abcb11*	Abcb11 ATP-binding cassette, sub-family B, member 11	−2.3 ± 0.26	11.6 ± 4.13
*Cdkn1a*	Cyclin-dependent kinase inhibitor 1A (p21, Cip1)	−1.9 ± 0.02	3.1 ± 0.19*
*Txnrd1*	Thioredoxin reductase 1	2.4 ± 0.44	1.3 ± 0.34*
*Slc39a6*	Solute carrier family 39 (zinc transporter), member 6	1.1 ± 0.68	2.1 ± 0.36*

Down-regulated genes			
*Cryl1*	Crystallin, lambda 1	−2.1 ± 0.39	1.2 ± 0.15
*Igfals*	Insulin-like growth factor binding protein, acid labile subunit	−2.1 ± 0.24	−1.1 ± 0.75
*Rdx*	Radixin	−1.3 ± 0.45	−2.1 ± 0.38*
*Krt18*	Keratin 18	1.1 ± 0.41	−2.2 ± 0.28*
*Slc2a3*	Solute carrier family 2 (facilitated glucose transporter), member 3	−2.3 ± 0.21	−1.1 ± 0.03
*Osta*	Organic solute transporter, alpha	−2.5 ± 0.22	−1.6 ± 0.23
*Casp3*	Caspase 3	−1.8 ± 0.37	−2.8 ± 0.6*
*Dnajb11*	DnaJ (Hsp40) homolog, subfamily B, member 11	−1.1 ± 0.49	−2.9 ± 0.27*
*Atp8b1*	ATPase, aminophospholipid transporter, class I, type 8B, member 1	−1.9 ± 0.26	−3.3 ± 0.26*
*Fasn*	Fatty acid synthase	−1.7 ± 0.10	−3.8 ± 0.12*
*Lss*	Lanosterol synthase (2,3-oxidosqualene-lanosterol cyclase)	−1.9 ± 0.20	−3.8 ± 0.55*
*S100a8*	S100 calcium binding protein A8	1.9 ± 0.68	−4.3 ± 0.57*
*Car3*	Carbonic anhydrase 3	−1.1 ± 0.28	−5.4 ± 0.98*
*Avpr1a*	Arginine vasopressin receptor 1A	−1.4 ± 0.19	−5.6 ± 0.42*
*Lgr5*	Leucine-rich repeat-containing G protein-coupled receptor 5	−1.1 ± 0.17	−6.1 ± 0.40*
*Srebf1*	Sterol regulatory element binding transcription factor 1	−1.2 ± 0.42	−6.3 ± 0.29*
*Cd36*	Fatty acid translocase	−1.1 ± 0.19	−7.9 ± 0.36*

Values shown are fold change ± SD; *n* = 3/group. Mice were exposed to BD for 6 hr/day, 5 days/week, for 2 weeks. Differential gene expression was determined by reverse-transcriptase qPCR as described in “Materials and Methods.”

* *p* < 0.05 compared with 6.25 ppm BD.

## References

[b1-ehp-119-635] Baccarelli A, Bollati V (2009). Epigenetics and environmental chemicals. Curr Opin Pediatr.

[b2-ehp-119-635] Bachman AN, Phillips JM, Goodman JI (2006). Phenobarbital induces progressive patterns of GC-rich and gene-specific altered DNA methylation in the liver of tumor-prone B6C3F1 mice. Toxicol Sci.

[b3-ehp-119-635] Bagnyukova TV, Tryndyak VP, Montgomery B, Churchwell MI, Karpf AR, James SR (2008). Genetic and epigenetic changes in rat preneoplastic liver tissue induced by 2-acetylaminofluorene. Carcinogenesis.

[b4-ehp-119-635] Barreto G, Schafer A, Marhold J, Stach D, Swaminathan SK, Handa V (2007). Gadd45a promotes epigenetic gene activation by repair-mediated DNA demethylation. Nature.

[b5-ehp-119-635] Benetti R, Gonzalo S, Jaco I, Schotta G, Klatt P, Jenuwein T (2007). Suv4-20h deficiency results in telomere elongation and derepression of telomere recombination. J Cell Biol.

[b6-ehp-119-635] Choi BM, Pae HO, Kim YM, Chung HT (2003). Nitric oxide-mediated cytoprotection of hepatocytes from glucose deprivation-induced cytotoxicity: involvement of heme oxygenase-1. Hepatology.

[b7-ehp-119-635] Cochrane JE, Skopek TR (1994). Mutagenicity of butadiene and its epoxide metabolites: I. Mutagenic potential of 1,2-epoxybutene, 1,2,3,4-diepoxybutane and 3,4-epoxy-1,2-butanediol in cultured human lymphoblasts. Carcinogenesis.

[b8-ehp-119-635] Couture JF, Collazo E, Ortiz-Tello PA, Brunzelle JS, Trievel RC (2007). Specificity and mechanism of JMJD2A, a trimethyllysine-specific histone demethylase. Nat Struct Mol Biol.

[b9-ehp-119-635] Dion V, Lin Y, Hubert L, Waterland RA, Wilson JH (2008). Dnmt1 deficiency promotes CAG repeat expansion in the mouse germline. Hum Mol Genet.

[b10-ehp-119-635] Esteve PO, Chin HG, Smallwood A, Feehery GR, Gangisetty O, Karpf AR (2006). Direct interaction between DNMT1 and G9a coordinates DNA and histone methylation during replication. Genes Dev.

[b11-ehp-119-635] Filser JG, Hutzler C, Meischner V, Veereshwarayya V, Csanady GA (2007). Metabolism of 1,3-butadiene to toxicologically relevant metabolites in single-exposed mice and rats. Chem Biol Interact.

[b12-ehp-119-635] Fodor BD, Kubicek S, Yonezawa M, O’Sullivan RJ, Sengupta R, Perez-Burgos L (2006). Jmjd2b antagonizes H3K9 trimethylation at pericentric heterochromatin in mammalian cells. Genes Dev.

[b13-ehp-119-635] Fuks F, Hurd PJ, Deplus R, Kouzarides T (2003). The DNA methyltransferases associate with HP1 and the SUV39H1 histone methyltransferase. Nucleic Acids Res.

[b14-ehp-119-635] Garzon R, Liu S, Fabbri M, Liu Z, Heaphy CE, Callegari E (2009). MicroRNA-29b induces global DNA hypomethylation and tumor suppressor gene reexpression in acute myeloid leukemia by targeting directly DNMT3A and 3B and indirectly DNMT1. Blood.

[b15-ehp-119-635] Goggin M, Swenberg JA, Walker VE, Tretyakova N (2009). Molecular dosimetry of 1,2,3,4-diepoxybutane-induced DNA-DNA cross-links in B6C3F1 mice and F344 rats exposed to 1,3-butadiene by inhalation. Cancer Res.

[b16-ehp-119-635] Goll MG, Bestor TH (2005). Eukaryotic cytosine methyltransferases. Annu Rev Biochem.

[b17-ehp-119-635] Greeson NT, Sengupta R, Arida AR, Jenuwein T, Sanders SL (2008). Di-methyl H4 lysine 20 targets the checkpoint protein Crb2 to sites of DNA damage. J Biol Chem.

[b18-ehp-119-635] Himmelstein MW, Acquavella JF, Recio L, Medinsky MA, Bond JA (1997). Toxicology and epidemiology of 1,3-butadiene. Crit Rev Toxicol.

[b19-ehp-119-635] Hsieh HC, Chen YT, Li JM, Chou TY, Chang MF, Huang SC (2009). Protein profilings in mouse liver regeneration after partial hepatectomy using iTRAQ technology. J Proteome Res.

[b20-ehp-119-635] Huang J, Wang Y, Guo Y, Sun S (2010). Down-regulated microRNA- 152 induces aberrant DNA methylation in hepatitis B virus-related hepatocellular carcinoma by targeting DNA methyltransferase 1. Hepatology.

[b21-ehp-119-635] IARC (International Agency for Research on Cancer) (2008). 1,3-Butadiene, Ethylene Oxide and Vinyl Halides (Vinyl Fluoride, Vinyl Chloride and Vinyl Bromide). IARC Monogr Eval Carcinog Risks Hum.

[b22-ehp-119-635] Jacinto FV, Esteller M (2007). MGMT hypermethylation: a prognostic foe, a predictive friend. DNA Repair (Amst).

[b23-ehp-119-635] Kemper RA, Krause RJ, Elfarra AA (2001). Metabolism of butadiene monoxide by freshly isolated hepatocytes from mice and rats: different partitioning between oxidative, hydrolytic, and conjugation pathways. Drug Metab Dispos.

[b24-ehp-119-635] Koc H, Tretyakova NY, Walker VE, Henderson RF, Swenberg JA (1999). Molecular dosimetry of N-7 guanine adduct formation in mice and rats exposed to 1,3-butadiene. Chem Res Toxicol.

[b25-ehp-119-635] Kojima M, Nemoto K, Murai U, Yoshimura N, Ayabe Y, Degawa M (2002). Altered gene expression of hepatic lanosterol 14α-demethylase (CYP51) in lead nitrate-treated rats. Arch Toxicol.

[b26-ehp-119-635] Kovalenko VM, Bagnyukova TV, Sergienko OV, Bondarenko LB, Shayakhmetova GM, Matvienko AV (2007). Epigenetic changes in the rat livers induced by pyrazinamide treatment. Toxicol Appl Pharmacol.

[b27-ehp-119-635] Lu K, Boysen G, Gao L, Collins LB, Swenberg JA (2008). Formaldehyde-induced histone modifications in vitro. Chem Res Toxicol.

[b28-ehp-119-635] Marlowe J, Teo SS, Chibout SD, Pognan F, Moggs J (2009). Mapping the epigenome—impact for toxicology. EXS.

[b29-ehp-119-635] Martens JH, O’Sullivan RJ, Braunschweig U, Opravil S, Radolf M, Steinlein P (2005). The profile of repeat-associated histone lysine methylation states in the mouse epigenome. EMBO J.

[b30-ehp-119-635] Melnick RL, Sills RC (2001). Comparative carcinogenicity of 1,3-butadiene, isoprene, and chloroprene in rats and mice. Chem Biol Interact.

[b31-ehp-119-635] Moggs JG, Orphanides G (2004). The role of chromatin in molecular mechanisms of toxicity. Toxicol Sci.

[b32-ehp-119-635] Morio LA, Leone A, Sawant SP, Nie AY, Parker JB, Taggart P (2006). Hepatic expression of heme oxygenase-1 and antioxidant response element-mediated genes following administration of ethinyl estradiol to rats. Toxicol Appl Pharmacol.

[b33-ehp-119-635] Peng JC, Karpen GH (2009). Heterochromatic genome stability requires regulators of histone H3 K9 methylation. PLoS Genet.

[b34-ehp-119-635] Peters AH, O’Carroll D, Scherthan H, Mechtler K, Sauer S, Schofer C (2001). Loss of the Suv39h histone methyltransferases impairs mammalian heterochromatin and genome stability. Cell.

[b35-ehp-119-635] Pogribny IP, Rusyn I, Beland FA (2008). Epigenetic aspects of genotoxic and non-genotoxic hepatocarcinogenesis: studies in rodents. Environ Mol Mutagen.

[b36-ehp-119-635] Pogribny IP, Tryndyak VP, Woods CG, Witt SE, Rusyn I (2007). Epigenetic effects of the continuous exposure to peroxisome proliferator WY-14,643 in mouse liver are dependent upon peroxisome proliferator activated receptor alpha. Mutat Res.

[b37-ehp-119-635] Pogribny I, Yi P, James SJ (1999). A sensitive new method for rapid detection of abnormal methylation patterns in global DNA and within CpG islands. Biochem Biophys Res Commun.

[b38-ehp-119-635] Rea S, Eisenhaber F, O’Carroll D, Strahl BD, Sun ZW, Schmid M (2000). Regulation of chromatin structure by site-specific histone H3 methyltransferases. Nature.

[b39-ehp-119-635] Reamon-Buettner SM, Mutschler V, Borlak J (2008). The next innovation cycle in toxicogenomics: environmental epigenetics. Mutat Res.

[b40-ehp-119-635] Sanders SL, Portoso M, Mata J, Bahler J, Allshire RC, Kouzarides T (2004). Methylation of histone H4 lysine 20 controls recruitment of Crb2 to sites of DNA damage. Cell.

[b41-ehp-119-635] Sawan C, Vaissiere T, Murr R, Herceg Z (2008). Epigenetic drivers and genetic passengers on the road to cancer. Mutat Res.

[b42-ehp-119-635] Schmittgen TD, Livak KJ (2008). Analyzing real-time PCR data by the comparative *C*_T_ method. Nat Protoc.

[b43-ehp-119-635] Shivapurkar N, Wilson MJ, Poirier LA (1984). Hypomethylation of DNA in ethionine-fed rats. Carcinogenesis.

[b44-ehp-119-635] Shuker DE (2002). The enemy at the gates? DNA adducts as biomarkers of exposure to exogenous and endogenous genotoxic agents. Toxicol Lett.

[b45-ehp-119-635] Swenberg JA, Ham AJ, Koc H, Morinello E, Ranasinghe A, Tretyakova N (2000). DNA adducts: effects of low exposure to ethylene oxide, vinyl chloride and butadiene. Mutat Res.

[b46-ehp-119-635] Tryndyak VP, Muskhelishvili L, Kovalchuk O, Rodriguez-Juarez R, Montgomery B, Churchwell MI (2006). Effect of long-term tamoxifen exposure on genotoxic and epigenetic changes in rat liver: implications for tamoxifen-induced hepatocarcinogenesis. Carcinogenesis.

[b47-ehp-119-635] Valinluck V, Sowers LC (2007). Endogenous cytosine damage products alter the site selectivity of human DNA maintenance methyltransferase DNMT1. Cancer Res.

[b48-ehp-119-635] Vire E, Brenner C, Deplus R, Blanchon L, Fraga M, Didelot C (2006). The Polycomb group protein EZH2 directly controls DNA methylation. Nature.

[b49-ehp-119-635] Wang J, Hevi S, Kurash JK, Lei H, Gay F, Bajko J (2009). The lysine demethylase LSD1 (KDM1) is required for maintenance of global DNA methylation. Nat Genet.

[b50-ehp-119-635] Weber M, Davies JJ, Wittig D, Oakeley EJ, Haase M, Lam WL (2005). Chromosome-wide and promoter-specific analyses identify sites of differential DNA methylation in normal and transformed human cells. Nat Genet.

[b51-ehp-119-635] White WC (2007). Butadiene production process overview. Chem Biol Interact.

[b52-ehp-119-635] Yamane K, Toumazou C, Tsukada Y, Erdjument-Bromage H, Tempst P, Wong J (2006). JHDM2A, a JmjC-containing H3K9 demethylase, facilitates transcription activation by androgen receptor. Cell.

[b53-ehp-119-635] Yang AS, Estecio MR, Doshi K, Kondo Y, Tajara EH, Issa JP (2004). A simple method for estimating global DNA methylation using bisulfite PCR of repetitive DNA elements. Nucleic Acids Res.

[b54-ehp-119-635] Yang H, Mizzen CA (2009). The multiple facets of histone H4-lysine 20 methylation. Biochem Cell Biol.

